# Electroacupuncture Relieves Neuropathic Pain via Adenosine 3 Receptor Activation in the Spinal Cord Dorsal Horn of Mice

**DOI:** 10.3390/ijms251910242

**Published:** 2024-09-24

**Authors:** Faisal Ayub Kiani, Hao Li, Sha Nan, Qiuhua Li, Qianghui Lei, Ruiling Yin, Shiya Cao, Mingxing Ding, Yi Ding

**Affiliations:** College of Veterinary Medicine, Huazhong Agricultural University, Wuhan 430070, China; drfakiani@webmail.hzau.edu.cn (F.A.K.); li_hao@webmail.hzau.edu.cn (H.L.);

**Keywords:** adenosine A3 receptors, analgesic effects, adenosine deaminase, electroacupuncture, neuropathic pain

## Abstract

Neuropathic pain (NPP) is a devastating and unbearable painful condition. As prevailing treatment strategies have failed to mitigate its complications, there remains a demand for effective therapies. Electroacupuncture (EA) has proved a potent remedial strategy in NPP management in humans and mammals. However, past studies have investigated the underlying mechanism of the analgesic effects of EA on NPP, focusing primarily on adenosine receptors in peripheral tissues. Herein, we elucidate the role of the adenosine (Adora-3) signaling pathway in mediating pain relief through EA in the central nervous system, which is obscure in the literature and needs exploration. Specific pathogen-free (SPF) male adult mice (C57BL/6 J) were utilized to investigate the effect of EA on adenosine metabolism (CD73, ADA) and its receptor activation (Adora-3), as potential mechanisms to mitigate NPP in the central nervous system. NPP was induced via spared nerve injury (SNI). EA treatment was administered seven times post-SNI surgery, and lumber (L4–L6) spinal cord was collected to determine the molecular expression of mRNA and protein levels. In the spinal cord of mice, following EA application, the expression results revealed that EA upregulated (*p* < 0.05) Adora-3 and CD73 by inhibiting ADA expression. In addition, EA triggered the release of adenosine (ADO), which modulated the nociceptive responses and enhanced neuronal activation. Meanwhile, the interplay between ADO levels and EA-induced antinociception, using an Adora-3 agonist and antagonist, showed that the Adora-3 agonist IB-MECA significantly increased (*p* < 0.05) nociceptive thresholds and expression levels. In contrast, the antagonist MRS1523 exacerbated neuropathic pain. Furthermore, an upregulated effect of EA on Adora-3 expression was inferred when the Adora-3 antagonist was administered, and the EA treatment increased the fluorescent intensity of Adora-3 in the spinal cord. Taken together, EA effectively modulates NPP by regulating the Adora-3 signaling pathway under induced pain conditions. These findings enhance our understanding of NPP management and offer potential avenues for innovative therapeutic interventions.

## 1. Introduction

Neuropathic pain (NPP) emerges as a consequence of nervous system dysfunction, presenting with aberrant sensory responses encompassing phenomena like hyperalgesia, allodynia, and spontaneous pain [1,2]. The initiation of pain perception within living organisms entails molecular mechanisms whereby nerve signals are transmitted and subsequently interpreted by the central nervous system (CNS) [3]. In the context of NPP, the upregulation of sodium channels and the release of neurotransmitters (glutamate and adenosine) play a pivotal role in neurotransmission. Furthermore, molecular aberrations in ion channel functions, neuronal structures, gene expressions, and neuroinflammation within neurons significantly contribute to neurodegenerative disorders [4]. NPP represents a substantial clinical challenge [5,6] because prevailing treatments (antidepressants and opioids) do not provide satisfactory relief.

Acupuncture, a component of traditional Chinese medicine, is among the innovative, efficacious, and safe interventions for the treatment of NPP [6]. Over a history spanning 3000–4000 years, acupuncture has been employed for pain management. Recently, electroacupuncture (EA), which combines electrical stimulation with traditional acupuncture to enhance efficacy and standardization, has demonstrated significant analgesic effects in various murine models of inflammatory, fibromyalgia, and neuropathic pain [7]. Furthermore, EA has been shown to alleviate multiple pain conditions by promoting the release of endogenous opioids [8] and increasing adenosine levels [9]. In addition, using EA demonstrates the notable potential in treating NPP [10,11]. Despite extensive utilization of EA, the exact mechanisms to alleviate NPP remained elusive.

Adenosine (ADO) is a critical neurotransmitter in pain management [12,13]. Biologically, ADO exerts its effect through four G protein-coupled ADO receptors (ARs; A1R, A2AR, A2B, and Adora-3), and their agonists have been previously used as potent antinociceptive in various pain models [14,15]. Among these, Adora-3 agonists (IB-MECA) are extensively used in the clinical conditions of psoriasis and rheumatoid arthritis [16,17,18,19]. Adora-3 is expressed in several CNS tissues, and its activation is essential for analgesia. It is also postulated that the EA procedure produces anti-nociceptive effects via ADO receptor-mediation [16]. Elevation in ADO levels within the localized Zusanli acupoint after traditional acupuncture treatment in mice was confirmed by localized administration of ADO receptor agonists [9]. Studies have investigated the mechanisms underlying the analgesic effects of EA, mainly focused on adenosine receptors and adenosine metabolism in peripheral tissues [20]. However, adenosine’s role in modulating neuropathic pain pathways, specifically the mechanism by which adenosine A3 acts within the CNS to mitigate neuropathic pain, requires further elucidation.

Adora-3 receptor activation in the context of EA, and its correlation in mediating anticonception in NPP, remain unexplored [21,22]. Therefore, the present study investigated the efficacy of EA in mitigating NPP and the underlying mechanisms involving Adora-3 receptor activation in the central nervous system. In addition, the impacts of an Adora-3 agonist and antagonist (IB-MECA and MRS1523, respectively) in NPP following EA treatment were determined to comprehensively underpin EA-induced analgesia via the Adora-3 pathway.

## 2. Results

### 2.1. EA Prompted Modifications in Adenosine Metabolism within the SCDH of SNI Mice

**Figure 1:**
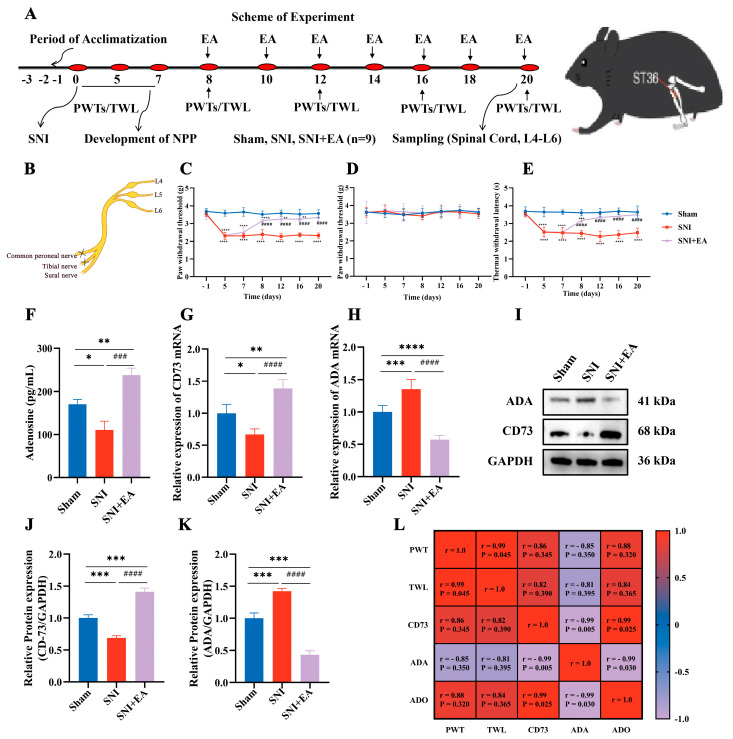
EA prompted modifications in adenosine metabolism within the SCDH of SNI mice. (**A**) The experimental layout and ST-36 acupoint in mice. (**B**) The methods used to create the SNI model. (**C**–**E**) The changes in Ipsilateral and Contralateral Paw Withdrawal Thresholds (g) and Thermal Withdrawal Latency (s) with and without EA treatment (n = 9). No significant difference was found in contralateral PWTs between the SNI, Sham, and SNI+EA groups. (**F**) Spinal adenosine levels (**G**,**H**), which depict EA and the upregulation of mRNA expression of CD73 while inhibiting the expression of ADA mRNA. (**I**–**K**) Western blotted protein bands, with GAPDH as an internal reference protein and relative protein expressions. The data are presented as the means ± SD. * Indicates statistical significance when compared with the sham group (*p* < 0.05). ** *p* < 0.01, *** *p* < 0.001, **** *p* < 0.0001 compared to the Sham group, ### *p* < 0.001, #### *p* < 0.0001 compared to the SNI group. (**L**) Pearson’s correlation (confidence interval: 95%) of CD-73 and ADA protein expressions with PWT, TWL.

The therapeutic impact of EA on mechanical and thermal hyperalgesia in mice with SNI-induced NPP was investigated (Figure 1C–E). Compared to the sham group, the SNI group exhibited a significant reduction (*p* < 0.001) in both paw PWT and TWL. However, following EA treatment, the SNI+EA group displayed a substantial increase in PWT and TWL in the ipsilateral hind paw on days 8, 12, 16, and 20 post-SNI surgery compared to the other groups. Notably, no significant change in PWT sensitivity was observed in the contralateral hind paw throughout the experimental period. Furthermore, the concentration of adenosine (ADO), assessed via ELISA on the 20th day post-surgery, was found to be decreased in the SNI group compared to the other groups. Conversely, EA treatment led to significantly higher levels (*p* < 0.05) of spinal ADO. Additionally, both mRNA and protein expressions of CD73 were significantly upregulated in the SNI+EA group compared to the sham and SNI groups (*p* < 0.05). In contrast, mRNA and protein expressions of ADA were notably higher in the SNI group compared to the sham or SNI+EA group. Interestingly, EA stimulation resulted in a significant decrease in ADA protein expression in SNI+EA mice, indicating a modulatory effect on ADO metabolism within the spinal cord dorsal horn (SCDH) as a therapeutic intervention for SNI-induced neuropathic pain. Moreover, correlation analysis between CD73, ADA, and ADO levels with PWT and TWL from the Sham, SNI, and SNI+EA groups revealed intriguing findings. Pearson’s correlation analysis demonstrated a strong positive correlation between nociceptive pain thresholds and CD73 and ADO levels. Conversely, a negative correlation was observed between ADA expression levels and nociception, further highlighting the regulatory role of these molecules in neuropathic pain. These results collectively underscore the efficacy of EA in modulating ADO metabolism and associated molecular pathways, thereby alleviating neuropathic pain symptoms.

### 2.2. Spinal Adenosine Is Necessary for the Anti-Nociceptive Effects of EA

**Figure 2:**
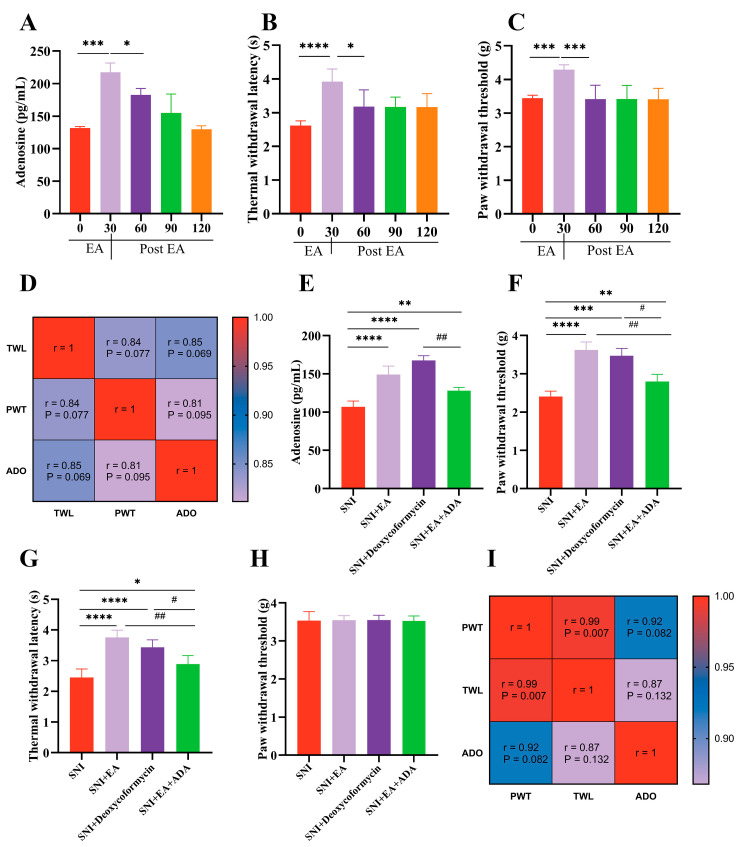
Electroacupuncture facilitated adenosine release, augmenting its analgesic efficacy. (**A**,**E**) The adenosine levels (pg/mL) in the spinal cords of wild-type and SNI mice were measured by ELISA (**B**,**C**), TWL, and PWTs before and after electroacupuncture at 0, 30, 60, 90, and 120 min. (**D**) Pearson’s correlation (confidence interval: 95%) of ADO with PWT, TWL at 0, 30, 60, 90, and 120 min (n = 6). (**F**,**G**) Paw withdrawal thresholds (PWTs) and thermal withdrawal latency (TWL) after 30 min of EA. (**H**) No difference in contralateral PWTs between SNI, SNI+EA, SNI+Deoxycoformycin, and SNI+EA+ADA. (**I**) Pearson’s correlation (confidence interval: 95%) of ADO with PWT, TWL between SNI, SNI+EA, SNI+Deoxycoformycin, and SNI+EA+ADA. The data are presented as the means ± SD. * Indicates statistical significance when compared with the control group # indicates statistical significance when compared with the SNI group # *p* < 0.05, ## *p* < 0.01, ** *p* < 0.01, *** *p* < 0.001, **** *p* < 0.0001 vs. the SNI group. ANOVA followed by Bonferroni’s post hoc test.

In wild-type mice, spinal adenosine levels significantly increased (*p* < 0.05) after 30 min of EA stimulation, gradually declining to baseline within 120 min, thereby elucidating the role of spinal adenosine in the anti-nociceptive effects of EA (Figure 2A). This release pattern was closely paralleled to nociceptive responses (Figure 2B,C), and PWT and TWL responses showed significant positive correlations (*p* < 0.05) with adenosine levels (Figure 2D). In an SNI mouse model, subsequent sampling was conducted 30 min post-EA treatment, the time point showing maximal adenosine concentrations in wild-type mice. Intrathecal injections of Deoxycoformycin and ADA on the eighth day post-SNI surgery demonstrated significantly elevated adenosine levels in the SNI +Deoxycoformycin group compared to other groups (*p* < 0.0001). Likewise, the SNI+EA and SNI+EA+ADA treatment groups showed increased adenosine levels (*p* < 0.001 and *p* < 0.01, respectively) compared to the SNI group (Figure 2E). However, the SNI+EA+ADA group exhibited reduced adenosine levels following ADA injection prior to EA treatment. Nociceptive thresholds revealed a significant increase in ipsilateral PWT (Figure 2F) and TWL (Figure 2G) following Deoxycoformycin and EA treatments compared to the SNI group. Notably, mice pretreated with ADA exhibited diminished antinociceptive effects of EA, while no significant changes were observed in the contralateral hind paw (Figure 2H). Correlation analysis between adenosine levels and pain thresholds revealed a positive correlation (*p* < 0.05) among the groups (Figure 2I).

### 2.3. Antinociceptive Effects of EA-Required Adenosine Adora-3 in Spinal Neurons

**Figure 3:**
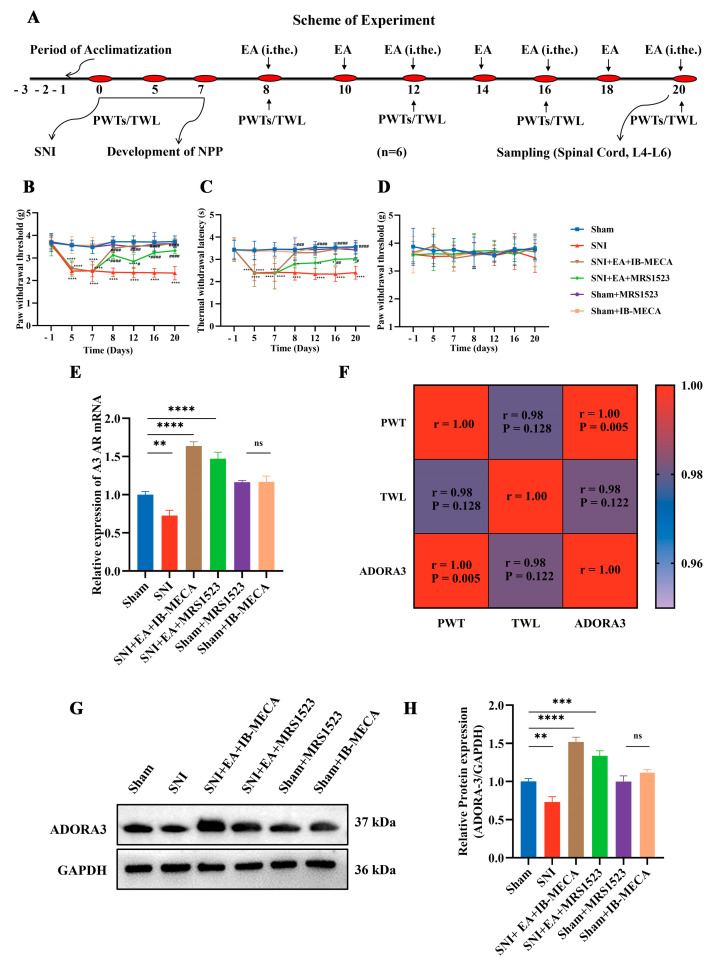
Adora-3 receptor agonist alleviates neuropathic pain after nerve injury and the anti-nociceptive effects of EA require Adora-3. (**A**) Scheme of experiment. (**B**,**C**) Ipsilateral paw withdrawal thresholds (PWTs) and thermal withdrawal latency (TWL). (**D**) Contralateral paw withdrawal thresholds (PWTs). (**E**) Relative mRNA expressions of Adora-3. (**G**,**H**) Relative expression levels of GAPDH, Adora-3, and western blotted bands. (**F**) Pearson’s correlation (confidence interval: 95%) between Adora−3 protein levels and PWT and TWL. Samples were collected from the L4–L6 segments of the spinal cord on day 20 post-surgery. The data are presented as means ± SD. On the same day, values with # indicate statistical significance when compared with the SNI group. * Indicates statistical significance when compared with the sham group (*p* < 0.05). ** *p* < 0.01, *** *p* < 0.001, **** *p* < 0.0001 compared to the Sham group, ## *p* < 0.01, ### *p* < 0.001, #### *p* < 0.0001 compared to the SNI group and ns indicates non-significant difference. ANOVA followed by Bonferroni’s post hoc test.

To investigate the specific role of Adora-3 in antinociception, the Adora-3 receptor agonist IB-MECA and antagonist MRS1523 were intrathecally administered to activate or inhibit the Adora-3 receptor, respectively. The baseline nociceptive thresholds remained stable in the sham groups throughout the study, while the SNI group exhibited a consistent decrease in nociceptive thresholds over time (Figure 3B,C). The mice administered with the Adora-3 receptor agonist exhibited a markedly elevated nociceptive threshold in ipsilateral paw withdrawal threshold (PWT) and thermal withdrawal latency (TWL). The ipsilateral PWT and TWL of the SNI+EA+IB-MECA group exhibited significantly higher values (*p* < 0.05) from day eight until the end of the experiment. The SNI+EA antagonist group showed a reduction (*p* > 0.05) in PWT and TWL compared to the SNI+EA+IB-MECA. The trend above suggests that the activation of the Adora-3 adenosine receptor through IB-MECA is implicated in regulating nociceptive thresholds induced by EA. This observation was subsequently corroborated by examining mRNA and relative protein expressions. However, there were no discernible differences in PWT among the groups for the contralateral hind paw, as shown in (Figure 3C). Likewise, the SNI+EA+IB-MECA group mRNA and the western blotted bands showed a significant (*p* < 0.05) difference in expression levels of Adora-3 compared to the other groups (Figure 3E,G,H). The relative expression levels of Adora-3 in the SCDH of antagonist groups were significantly lower (*p* > 0.05) compared to the agonist groups. A progressive increase in Adora-3 receptor mRNA and relative protein levels over the observation period was noted in the SNI group following intrathecal administration of IB-MECA in the EA group. On the other hand, administering the Adora-3 antagonist MRS1523 to the SNI+EA mice resulted in a downregulation of Adora-3 mRNA and protein expression. Pearson’s correlation analysis between Adora-3 expression levels and PWT, TWL in the SNI, SNI+EA+IB-MECA, and SNI+EA+MRS groups is shown in Figure 3F. The Adora-3 protein expression levels were positively correlated with PWT and TWL.

### 2.4. EA Enhanced the A3 Receptor Florescence Intensity within the Spinal Neurons of SNI Mice

**Figure 4:**
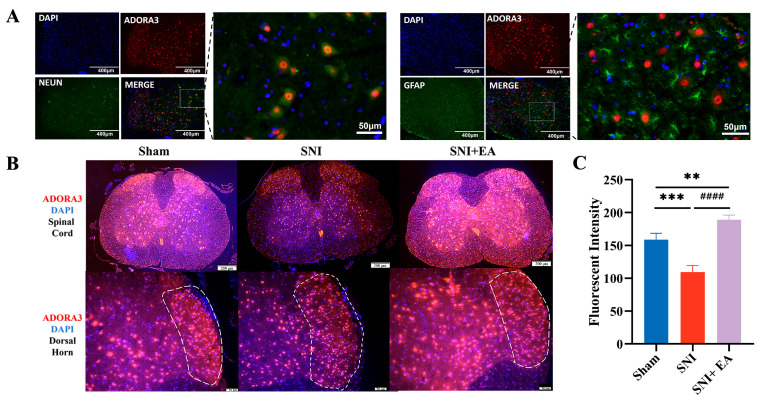
EA enhanced the Adora-3 receptor fluorescence intensity within the spinal neurons of SNI mice (**A**,**B**). Colocalization and distribution of Adora-3 with the marker of neurons and astrocytes in the SCDH. (**C**) Quantification of the mean fluorescence intensity of Adora-3 in the SCDH. Scale bars of 400 μm, 200 μm, and 50 μm were employed. Samples were collected from the L4–L6 segments of the spinal cord on day 20 post-surgery. The data are presented as the means ± SD. ** *p* < 0.01, *** *p* < 0.001, compared with the normal group, #### *p* < 0.001 compared with the SNI group. n = 3 in all groups. One-way ANOVA followed by Bonferroni’s post hoc test.

To investigate the impact of Adora-3 receptor activation on antinociception, a detailed examination of its distribution in the neurons of the SCDH was performed. Figure 4A illustrates the spatial distribution and co-localization of Adora-3 with neuronal marker neuronal nuclear antigen (Neun) and astrocyte marker glial fibrillary acidic protein (GFAP). Adora-3 immunoreactivity exhibited a high co-expression with Neun and sparse co-expression with GFAP through double-immunofluorescence staining. IF staining revealed that Adora-3 predominantly localized within neurons in the SCDH of mice. Compared to the Sham group, the reduced fluorescence intensity of Adora-3 was observed in the SCDH of the SNI group, which was reversed by EA treatment (Figure 4C). Moreover, when comparing groups without EA, EA significantly enhanced (*p* < 0.05) the distribution and fluorescent intensity of Adora-3 in the SCDH of mice (Figure 4B).

## 3. Discussion

Neuropathic pain (NPP) following nerve injury makes the life of patients extremely painful [23] and remains a challenging condition to manage, necessitating innovative therapeutic approaches. Pharmacological approaches are commonly used for temporary relief [24]. However, prolonged use of drugs usually leads to adverse reactions or drug tolerance. Hence, developing novel targeted medicines or other interventional measures is crucial to alleviate the suffering of NPP patients [25,26]. In this study, we investigated the potential of electroacupuncture (EA) and Adora-3 in mitigating NPP as novel approaches and therapeutic targets. Our comprehensive investigation encompassed multiple facets of the impact of EA, its effects on nociceptive thresholds, ADO modulation, neuronal activation, and Adora-3 receptor expression using the SNI model.

The therapeutic effects of acupuncture, including EA, have long been associated with regulating the nervous system at multiple levels, including the peripheral, spinal, and supraspinal domains [27,28]. The SCDH emerges as a crucial site for pain modulation and is located within the region where peripheral nerves convey sensory information to the brain through projection neurons [29]. The current data suggest that nociceptive hyperalgesia within the CNS is orchestrated by intricate neuronal events within the SCDH [30]. The SCDH was chosen over the dorsal root ganglion for investigating Adora3 expression because it is a crucial site for central nociceptive processing, exhibits significant expression of adenosine receptors, serves as a target for pain therapeutics, and plays an essential role in sensory integration [31]. It receives direct input from primary sensory neurons, including those in the DRG, and plays a pivotal role in integrating and transmitting pain signals to higher brain centers [32]. The mechanism involved in EA-mediated pain relief is an intricate system of neurotransmitters and neuromodulators within the peripheral and CNS. This network encompasses neurotransmitters, including norepinephrine, glutamate, γ-aminobutyric acid, opioid peptides, ADO, and serotonin [33]. Among these substances, adenosinergic signaling plays a pivotal role in shaping somatosensory sensations within the SCDH under physiological and pathological conditions, affecting neuronal activation and adenosine receptor expression [34]. In this study, we selected Adora-3 over other ADO receptors because of its safety profile [19], as other receptors elicit untoward effects on the cardiovascular system besides analgesic effects [15]. In our study, we employed the SNI mouse model and followed the protocol established previously [35], which effectively allowed us to investigate the impact of EA on NPP. The present study is the first of its kind to elucidate that the Adora-3 receptor mediates the antinociception of EA to relieve NPP in the SCDH.

The current investigations determined that the SNI mice exhibited prolonged somatosensory hypersensitivity and abnormal pain. However, following EA treatment, the mice displayed a significant increase in both mechanical and thermal nociceptive thresholds, indicating a noteworthy analgesic effect of EA, which is in line with prior findings using a similar model [36,37]. Our findings demonstrated that adenosine (ADO) concentrations were significantly elevated in both wild-type and SNI mice following EA stimulation, indicating a substantial increase in spinal ADO levels. Moreover, the administration of the ADA inhibitor deoxycoformycin significantly impacted ADO concentration and analgesic effects. These findings highlighted notable correlations between ADO release levels and changes in nociceptive thresholds, thereby validating the effects of EA in our experiment. Additionally, ADA treatment inhibited ADO, consequently attenuating the analgesic efficacy of EA, as observed previously [9]. ADO plays a pivotal and indispensable role in mediating the analgesic effects of EA, as evidenced by previous findings [38] which demonstrated that EA upregulated ADO levels in the L4–6 spinal cord of CCI+EA rats. These findings potentially underscore the involvement of ADO in the amelioration of NPP, demonstrating an essential facet of EA as a therapeutic approach. In our current investigation, we also optimized the exact time of maximum ADO release following EA treatment. We found the peak ADO release approximately 30 min after EA shots, which is consistent with earlier results in which ADO concentration increased almost 24-fold during the 30 min of the acupuncture session [9]. The persistence of prolonged antinociceptive effects of the Adora-3 agonist [18,39] did not result in tolerance development to Adora-3 agonism. Considerably elevated mechanical and thermal nociceptive thresholds and higher expressions of Adora-3 mRNA and protein in the SCDH of mice treated with the selective Adora-3 agonist IB-MECA indicate the role of the Adora-3 receptor as a nociception inhibitor. Similar findings were documented earlier [18], in a study that investigated the locations and neurological mechanisms underpinning the antinociceptive effects of Adora-3 activation. During NPP, spinal nociceptive processing is accelerated by the increased spontaneous activity of wide-range pain projection neurons and controlled by both spinal and supraspinal (e.g., RVM) mechanisms [40]. Similarly, the current data also showed the relative protein and mRNA expressions of Adora-3 protein in the SCDH. In contrast, when MRS1523 was injected into the spinal cord, it reduced Adora-3 levels. This suggests that the Adora-3 receptor is activated by IB-MECA in the spinal cord, confirming its presence in areas related to nociception in the central nervous system. It shows the role of Adora-3 in ADO-mediated nociceptive relief; therefore, the intrathecal IB-MECA had sufficiently induced antinociception in SNI mice, corroborating earlier findings [33]. This expression pattern suggests the analgesic effects of EA linked to long-term regulation of the spinal Adora-3 pathway. Likewise, EA treatment attenuated the CCI-induced nociception at the level of SCDH, suggesting a significant role of EA after CCI. Likewise, EA regulated the level of CREB in mice’s ACCs and spinal cords [41].

The intrathecal administration of the Adora-3 antagonist MRS1523 inhibited Adora-3, thereby diminishing the analgesic effect of EA. This suggests that MRS1523 impacted endogenous ADO levels and downregulated Adora-3 expression in the spinal cord of SNI mice. The reduced nociceptive thresholds following intrathecal MRS1523 in the SNI+EA group highlight MRS1523’s role in partially reversing the analgesic effect of EA. These findings illustrate the analgesic roles of ADO and Adora-3 in the L4–6 spinal segments under SNI conditions. Immunofluorescence staining revealed that Adora-3 was highly colocalized with the neurons of the SCDH. Immunofluorescence staining confirmed the colocalization patterns of Adora-3, suggesting the participation of spinal Adora-3 in the EA-evoked attenuation of SNI-induced NPP. These findings align with a previous study [38] where EA-related activation of the A1 AR signaling pathway in the pathogenesis of NPP was observed. EA has been found to increase the fluorescent intensity of Adora-3 in the present study. In another study, it was observed that Adora-3 was expressed in the brain, and the cellular colocalization of Adora-3 was found in neurons [42]. Similar results were observed in other animal pain models when A1 Rs in the spinal cord were stimulated for EA-induced analgesia [43]. These studies suggest an underlying mechanism for EA-induced increases in ADO levels in the L4–6 spinal segments, which may involve the release of ATP within neurons or spinal neuronal cells into the extracellular space, which is then converted into ADO by ATP lyase and other enzymes. Furthermore, another study [9] found the analgesic effect of acupuncture is associated with the release of ADO in peripheral tissues and suggested acupuncture’s role in releasing ATP in tissues for subsequent metabolism into ADP, AMP, and ADO. AMP can be retained in tissues for longer periods before being gradually metabolized to ADO [44]. Based on current data, it is hypothesized that the analgesic effect of EA is linked to an increase in ADO levels and the accumulation of AMP in tissues for up to 30 min following an EA session; however, this hypothesis needs to be confirmed by measuring AMP levels in tissues after single or multiple EA sessions.

To sum up, this study primarily aimed to investigate the impact of EA on the Adora-3 receptor and its related factors in modulating NPP in the SCDH of treated mice groups within the CNS. However, the findings of this study serve as a baseline for numerous avenues of research. One such possibility is the extension of this research investigation to supraspinal tissues in the CNS, including non-human primates and large mammals, to gain a more comprehensive understanding of the subject. Moreover, the natural progression of this line of investigation should eventually extend to human subjects to explore the synergistic effects of EA when combined with other interventions such as pharmaceuticals or physical therapies.

## 4. Materials and Methods

### 4.1. Experimental Animals and Management

Specific pathogen-free male adult C57BL/6J mice, provided by the Huazhong Agricultural University Laboratory Animal Center, were utilized in this study. These mice weighed between 20 and 25 g and were aged between 8 and 12 weeks. The experimental procedures were approved by the Institute of Animal Care and Use Committee of Huazhong Agricultural University, Wuhan, China (Permit no. HZAUMO-2024-0005). The mice were given one week to acclimate before the commencement of the experimental procedures. They were housed under controlled conditions, maintaining a temperature of 23 °C ± 1 °C and a 12 h light/dark cycle. Additionally, the mice had unrestricted access to food and water throughout the study.

### 4.2. Induction of the Neuropathic Pain

Before the surgical intervention for SNI modeling, the mice underwent a 24 h fasting period. The SNI was employed to induce NPP, following established protocols [35]. Briefly, the mice were first subjected to anesthesia with 1% sodium pentobarbital [45]; subsequently, the surgical site was shaved and disinfected. A minor longitudinal incision, 0.5–1 cm in length, was carefully made on the posterior–lateral aspect of the thigh to access the sciatic nerve and its three principal branches (the common peroneal, the tibial, and the sural nerves). The tibial and common peroneal nerves were ligated using 5-0 silk followed by a distal cut of 2 mm to the ligature, and the sural nerve was preserved. Afterward, the muscles were sutured using a single muscle suture, and the skin was closed through a skin clip.

Meanwhile, the mice in the sham group underwent identical surgical procedures without nerve ligation. The surgical procedure was completed within the stipulated time to minimize potential cognitive impact. The integrity of the sciatic nerve in the sham group was evaluated through gentle tapping and tugging [46]. After a seven-day interval following SNI surgery and the establishment of NPP, the treated mice groups were subjected to EA sessions, as well as the administration of agonists and antagonists, according to the scheduled period outlined in Figure 1 and Figure 3A.

### 4.3. Intrathecal Injection

In the present study, an Adora-3 agonist (IB-MECA, N6-(3-iodobenzene)-adenosine-5-N-methyluronamide) and Adora-3 antagonist (MRS1523,3-propyl-6-ethyl-5(ethylthio)carbonyl-2-phenyl-4-propyl-3-pyridine carboxylate) (MCE, Shanghai, China) were utilized. To prepare stock solutions (10 mM), the agents were dissolved in DMSO [47] and stored at −80 °C until needed. Deoxycoformycin and ADA (GLP.BIO., Montclair, CA, USA) were dissolved in sterile endotoxin-free isotonic saline. Later, the IB-MECA and MRS1523 were administered at doses of 60 and 3 nmol intrathecally (i.th.) on the 8th, 12th, 16th, and 20th days of NPP induction, as recommended [48]. Deoxycoformycin and ADA were given intrathecally before the EA sessions [49,50,51]. For intrathecal injections, the mice were shaved at the lumbar region, sterilized, and gently secured using a cotton cloth for safety and calmness. To achieve a slight curvature of the spine at the L4–L6 vertebrae level, a centrifuge tube (approximately 15 mL) was placed under the abdominal-pelvic area, applying pressure to stabilize the sacrum and locate the L4–L6 vertebrae using the anterior iliac crest as a reference. Subsequently, a 30-G guide needle was inserted into the intervertebral space. Prior to delivering drugs into the cerebrospinal fluid through the guide needle, a catheter (PE-10) was marked to rest above the L4–L6 lumbar spinal cord. A volume of 2 µL of sterile saline was infused to ensure thorough medication distribution intrathecally. The intrathecal injection lasted approximately 20 s, followed by a 30 s pause before removing the catheter and guiding the needle [49]. The flick of the tail served as an indication of the entrance of the needle to the subarachnoid space. 

### 4.4. EA Treatment

The mice were restrained in a specially designed mouse fixer to facilitate EA sessions and underwent a 30 min acclimatization period. This process was practiced daily for at least three days preceding the formal EA procedure. For EA sessions, the bilateral acupoints “Zusanli” (ST-36) [52], known for anti-inflammatory [53] and immune-enhancing [54] properties, were chosen, and these are located approx. 3–4 mm below and 1–2 mm lateral to the knee’s midline [27], as depicted in Figure 1A. ST36 is vital due to its high therapeutic efficiency in various diseased conditions, including NPP [55]. Stimulation of ST36 produces an antinociceptive effect via neuronal transmission in the spinal dorsal horn [56]. Considering ST-36 as the therapeutic acupoint for mice experiencing NPP, the mice were treated for seven EA sessions at 48 h intervals, commencing on the eighth day after the SNI intervention. A specially designed electric stimulation device (Hans-200e, Jisheng Medical, Beijing, China) was employed to administer continuous-mode stimulation at the ST36 acupoint. The electric stimulus was administered at a frequency of 2 Hz with a current of 2 mA for 30 min, as suggested [57]. To enable this process, pairs of stainless steel unipolar acupuncture needles (0.25 × 30 mm, Dongbang Acupuncture, Inc., Seoul, Republic of Korea)were interleaved into the ‘ST36’ point at a depth of 2 mm, positioned 3–4 mm below and 1–2 mm across from the knee midline [58]. Throughout this procedure, it was ensured that the mice were entirely calm without displaying any signs of distress.

### 4.5. Nociceptive Behavior Tests

To observe nociception, the sensory nervous system encodes painful stimuli [59], which involves detecting, converting, and recognizing painful signals. Nociception is triggered by mechanical and thermal stimulation of sensory neurons, transmitting signals through nerve fibers to the brain via the spinal cord. The nociceptive pain was observed through the following behavioral tests.

#### 4.5.1. Paw Withdrawal Threshold (PWT)

The mechanical threshold, PWT, was assessed three days before the SNI surgery (baseline threshold value) and at four intervals after seven days post-surgery. Mice were placed in a transparent plexiglass box with a metal mesh pad (5 × 5 mm^2^ mesh area) at the bottom for a 30 min adaptation period. Subsequently, a series of electronic Von Frey filament anesthesiometer forces (ZS-Dichuang Science and Technology Development Co., Ltd., Beijing, China) were applied perpendicularly to the plantar surface of the hind paw for 6 s [60]. The range of Von Frey filament forces spanned from 0.07 to 1.4 g. A positive response was recorded if the animal licked its feet or withdrew its paw. The mechanical stimulus was discontinued upon hind paw withdrawal, and the force was noted as PWT. This test was observed three times at 5 min intervals.

#### 4.5.2. Thermal Withdrawal Latency (TWL)

To assess TWL, the Hot/Cold Plate Analgesia Meter (Zhongshi-Dichuang Science and Technology Development Co, Ltd., Beijing, China) was used, equipped with a metal plate with a temperature range from −3 (cold) to 65 °C (hot), while maintaining an ambient temperature of from 20 to 25 °C. The electronic thermostat regulates the plate’s temperature, and a digital thermometer on the front panel shows the temperature of the current plate. Pain sensitivity from exposure to heat was assessed by placing the mice on the plate surface and initiating a built-in timer. The operator stopped the timer when the mice raised the paw from the plate in response to cold or hot temperatures [61]. The front panel timer displayed the duration in seconds for the mice to react or withdraw their paw in response to the stimulus, with reaction time recorded as a measure of pain thresholds. Each mouse underwent pain-related behavioral assessments at five-minute intervals, with a total of five evaluations.

### 4.6. Collection of Samples

To investigate the central regulatory mechanism of EA in the management of NPP, and to analyze the expression levels of Adora-3, spinal cord samples were collected according to the treatment protocol. The spinal cord within the lumbar vertebrae region (L4–L6) was meticulously dissected; for immunofluorescence analysis, samples were preserved in formalin, whereas, for ADO levels, samples were immediately processed for determination of spinal adenosine concentration through Elisa ADO kit according to manufacturer’s instructions. The spinal cord was instantly frozen in liquid nitrogen and stored at −80 °C for western blotting and RT-PCR analysis.

### 4.7. Animal Grouping

To explicate the effect of electroacupuncture on adenosine metabolism in mitigating the NPP induced by SNI, the selected twenty-seven mice were divided into sham (n = 9), SNI (n = 9), and SNI+EA (n = 9) groups, respectively. This study evaluated the ADO-metabolizing enzymes, CD73 and ADA, using RT-qPCR and western blotting on day 20 post-surgery to investigate the effects of EA. Adenosine metabolism is regulated by both 5′-nucleotidase (CD73) and adenosine deaminase (ADA) enzymes [62,63]. EA treatment was administered seven times at 8, 10, 12, 14, 16, 18, and 20 days post-SNI surgery (Figure 1A). Mechanical and thermal sensitivities were recorded three times before and on days 8, 12, 16, and 20 of SNI surgery, 30 min after each EA session. After measuring nociceptive thresholds on the seventh EA session, the spinal cord samples were collected for immunofluorescence staining (3 samples/group) and molecular investigations (6 samples/group) on day 20 of SNI surgery. To assess the extracellular ADO concentration before and after EA treatment, wild-type mice (n = 24) were used. The mice were divided into a control (no EA treatment) and four EA treatment groups (according to sampling times 30, 60, 90, and 120 min following EA treatment). The mice (n = 3/group) were euthanized and immediately sampled for the spinal cord, following EA treatment at their respective time intervals for determination of spinal ADO through ELISA ADO KIT. The remaining mice in each group underwent nociceptive threshold assessments at the designated time points. To observe the effect of EA on ADO concentration following NPP in SNI, the mice were randomly allocated into SNI, SNI+EA, SNI+Deoxycoformycin, and SNI+EA+ADA groups. The SNI+Deoxycoformycin group was given deoxycoformycin (10 μM; an adenosine metabolism inhibitor) intrathecally during EA treatment. In contrast, SNI+EA+ADA mice received an intrathecal injection of ADA (0.5 U) 30 min before the onset of the EA procedure. Mechanical and thermal nociceptive threshold measurements and spinal cord sampling were performed after 30 min of EA sessions. Subsequently, the ADO contents were assessed in the samples using the ELIZA ADO KIT, following the manufacturer’s instructions. The effect of Adora-3 receptor activation in EA-induced antinociception was investigated by intrathecal injection of Adora-3 agonist 30 min before the EA session. Adora-3 receptors agonist and antagonist were administered intrathecally to the experimental groups (Sham, SNI, SNI+EA+IB-MECA, SNI+EA+MRS1523, Sham+MRS1523, and Sham+IB-MECA, n = 6/group) on 8, 12, 16, and 20 days post-SNI surgery (Figure 3A). Subsequently, mechanical and thermal hypersensitivities were measured, and spinal cord samples were collected for molecular analysis on day 20. To elucidate the expression and distribution levels of Adora-3 in the SCDH, double-labeling immunofluorescent staining was performed on the samples collected from the ADO metabolism studies. This immunofluorescent staining facilitated the observation of Adora-3 colocalization with the neuronal marker Neun and the astrocyte marker GFAP (Figure 4A–C).

### 4.8. Immunofluorescence Staining (IF)

Following extraction of the spinal cord (L4–L6), the samples were fixed in a 4% paraformaldehyde solution for 2 h. The samples were then embedded in paraffin, and each block was consecutively sectioned at a thickness of 5 µm. The paraffin slides holding spinal cord tissue were incubated for 2 h at 37 °C and subsequently dewaxed through a series of solutions: Xylene I for 15 min, Xylene II for 15 min, Xylene III for 10 min, 100% Ethanol I for 3 min, 100% Ethanol II for 3 min, 95% Ethanol for 3 min, 80% Ethanol for 3 min, and 75% Ethanol for 3 min. The slides were then washed three times with PBS. Next, the slides were immersed in a citrate repair solution and heated using a steamer to 92–98 °C, maintained for 20 min, and allowed to cool to room temperature naturally. After cooling, the slides were washed with PBS for 5 min three times. A 5% BSA blocking solution was then added to the tissues, which were incubated at 37 °C for an hour before being washed with PBS. The sections were subsequently treated with an Adora-3 polyclonal antibody (Thermo Fisher Invitrogen, Waltham, MA, USA, diluted 1:200), an anti-NeuN antibody (Proteintech 66836-1-Ig, Rosemont, IL, USA, diluted 1:200), and an anti-GFAP antibody (Cell Signaling Technology (CST), Danvers, MA, USA, 66836-1-Ig, diluted 1:200) at 4 °C overnight. After washing with PBS, the sections were treated with Cy3 or FITC-labeled anti-rabbit IgG (Servicebio, GB121152; Wuhan, China) at the appropriate dilution, incubated for 30 min at 37 °C, and washed with PBS. Preferential incubation for Adora3 was performed with stable red Cy3 (1:1000).

The slides were then counterstained with DAPI (Beyotime, C1002, Shanghai, China) for 10 min at room temperature, washed, and mounted with an antifade medium to protect them from light. Images were captured using a (4× and 20×) objective fluorescent microscope (Olympus, BX-51, Tokyo, Japan). Red fluorescence represented Adora-3, green fluorescence indicated neurons or astrocytes, blue fluorescence represented the cell nucleus, and yellow fluorescence indicated the colocalization of Adora-3 with NeuN or GFAP. Quantitative analysis of signal intensity was conducted using ImageJ software (version 1.53, NIH, Bethesda, MD, USA).

### 4.9. Real-Time Quantitative PCR

The gene expression analysis via the rt-qPCR method involved extracting total RNA from spinal cord tissue samples (L4–L6) using TRIzol Reagent (TaKaRa Bio, Inc., Dalian, China). RNA concentrations were normalized and reverse transcribed into cDNA using the Superscript II First-Strand Synthesis System (ABclonal Biotechnology, Wuhan, China). cDNA synthesis was carried out with random hexamer primers and a dNTP mix, followed by treatment with a reverse transcriptase enzyme. The resulting cDNA was stored at −80 °C until RT-qPCR analysis. Primer sequences were obtained from NCBI GenBank (Table 1) (https://www.ncbi.nlm.nih.gov/genbank/ accessed on 15 March 2023).

; PCR amplification was performed using a Quantitect SYBR Green PCR kit on a MyiQ Single Color Real-Time PCR Detection System. Reaction mixtures contained Quanti-Tect SYBR Green (QIAGEN, Hilden, Germany), forward and reverse primers, nuclease-free water, and cDNA from each sample. Amplification conditions included initial denaturation, denaturation, annealing, and extension. Cq values were recorded for each reaction. Each biological sample was run in triplicate (technical replicates), and three biological replicates were included per condition. Data were analyzed using the ΔΔCT method [64], and results were normalized to GAPDH expression. The relative mRNA quantification was standardized using glyceraldehyde-3-phosphate dehydrogenase (GAPDH) as a reference gene, and the fold change in expression was calculated using the ΔΔCT method.

### 4.10. Western Blotting

The western blotting analysis determined the Adora-3 at the protein expression level. Initially, tissue samples were homogenized in a 100:1 lysate solution (RIPA: PMSF) and centrifuged at 12,500 rpm for 6 min at 4 °C. The protein content was determined using a BCA protein assay kit (Beyotime Corp., Shanghai, China) and placed onto a Tris HCl SDS-PAGE gel (Bio-Rad Lab., Hercules, CA, USA) for electrophoresis at 80 V. Afterwards, PVDF membranes (Millipore Corp., Burlington, MA, USA) were used to transfer the proteins. The membranes were blocked for two hours with 5% skim milk and were treated with primary antibodies (Adora-3, Affinity Biosciences: DF4851, Cincinnati, OH, USA, diluted 1:500), a CD-73 polyclonal antibody (12231-1-AP, Proteintech, Rosemont, IL, USA, diluted 1:1000, an ADA polyclonal antibody (13328-1-AP, Proteintech, Rosemont, IL, USA), diluted 1:500), and GAPDH (AB-clonal, AC001, Wuhan, China, 1:3000 diluted in 5% BSA) at 4 °C overnight. After washing, the membranes were exposed to secondary antibodies (Donkey anti-rabbit-IgG, AB-clonal, AS038, Wuhan, China; 1:50,000 in TBST) for 2 h. Imaging was performed using a UVP gel imaging apparatus (Upland, CA, USA), and data analysis was conducted using ImageJ software (Version 1.53, N.I.H, Bethesda, MD, USA).

### 4.11. ELISA

The levels of ADO contents in the spinal cord were measured by the ELISA ADO kit (Fk2908, Jiangsu Jingmei Biological Technology Co., Ltd., Yancheng, China), following the manufacturer’s instructions.

### 4.12. Statistical Analysis

For statistical analysis, GraphPad Prism version 8.0.1, San Diego, CA, USA was used. The mean ± standard deviation (SD) was used to present each observation. The nociceptive behavioral data of ipsilateral and contralateral sides were examined separately. Following data normalization, the nociceptive measurements were subjected to statistical analysis model repeated-measures two-way ANOVA, considering different treatments as primary variables. One-way ANOVA was employed for values obtained through RT-qPCR. The statistical significance between the variables was determined using the Bonferroni post hoc test (*p*-value < 0.05).

## 5. Conclusions

In conclusion, electroacupuncture (EA) emerged as a viable alternative to chemical therapies for pain management, effectively mitigating NPP by modulating the expression of Adora-3, CD73, and ADA in the spinal cord of mice. Furthermore, EA significantly attenuates NPP through a rapid increase in adenosine (ADO) levels upon stimulation. The analgesic effects of EA are mediated by the upregulation of Adora-3 and CD73 and the inhibition of ADA expression in the spinal cord.

## Figures and Tables

**Table 1:** Primer sequences.

Gene	Sequence (5′->3′)	Gene Bank Accession No.
Adora-3	F: GGAAGCCGACAACACCACG-3	NM_009631.4
	R: CTTGACCACCCAGATGACCAG	
CD-73	F: AGCGATGACTCCACCAAGIG-3	NM_011851.4
	R: CAGATGGTGCCCTGGTACIG-3	
ADA	F: CGACTGAACATCAACGCAGC-3	NM_001272052.1
	R: TCAGTCTGTGGTGGCTATTGG-3	
GAPDH	F: TGAGCCTCCTCCAATTCAACC-3	NM_0011289726.2
	R: AATCCGTTCACACCGACCTT-3	

## Data Availability

The datasets used and/or analyzed during the current study are available from the corresponding author upon reasonable request.
